# The order of complexity of visuomotor learning

**DOI:** 10.1186/s12868-017-0368-x

**Published:** 2017-06-12

**Authors:** John Kim, Fariya Mostafa, Douglas Blair Tweed

**Affiliations:** 1 Department of Physiology, University of Toronto, Toronto, ON M5S1A8 Canada; 2 Centre for Vision Research, York University, Toronto, ON M3J1P3 Canada; 3 College of Medicine, University of Manitoba, Winnipeg, MB R3E 3P5 Canada

**Keywords:** Learning algorithms, Adaptive control, Sensorimotor systems

## Abstract

**Background:**

Learning algorithms come in three orders of complexity: zeroth-order (perturbation), first-order (gradient descent), and second-order (e.g., quasi-Newton). But which of these are used in the brain? We trained 12 people to shoot targets, and compared them to simulated subjects that learned the same task using various algorithms.

**Results:**

Humans learned significantly faster than optimized zeroth-order algorithms, but slower than second-order ones.

**Conclusions:**

Human visuomotor learning is too fast to be explained by zeroth-order processes alone, and must involve first or second-order mechanisms.

**Electronic supplementary material:**

The online version of this article (doi:10.1186/s12868-017-0368-x) contains supplementary material, which is available to authorized users.

## Background

When you learn a visuomotor task, such as shooting a target, how do your neural circuits know which synaptic changes will make you more accurate? We know a great deal about the biochemistry of synaptic change, both strengthening and weakening, but much less about how synapses decide, based on feedback from the senses, whether to strengthen or to weaken, and by how much. The simplest possibility is that the brain adjusts its synapses randomly, then uses sensory feedback to detect whether performance has improved, and retains or undoes its adjustments on that basis—a method known as *zeroth*-*order* (or correlation or perturbation) learning [1–3]. Or, the brain may learn more efficiently by computing non-random adjustments designed to enhance performance. It may compute a promising direction of synaptic change—a method called *first*-*order* (or gradient descent) learning [2, 4, 5]. Or it may compute more-effective changes by *second*-*order* (or quasi-Newton or Hessian-free) learning, though these methods call for more complex networks [2, 6].

Many neuroscientists have suggested that the brain may use zeroth-order learning, owing to its simplicity [1, 7–10], but others have proposed first-order schemes [11–15]. To test these ideas, we had 12 human subjects learn a visuomotor task, and raced them against computer-simulated zeroth, first, and second-order learners. We found that human learning was too fast to be explained by zeroth-order learning alone, and so must incorporate first or second-order mechanisms.

## Results

### Human learning

**Fig. 1:**
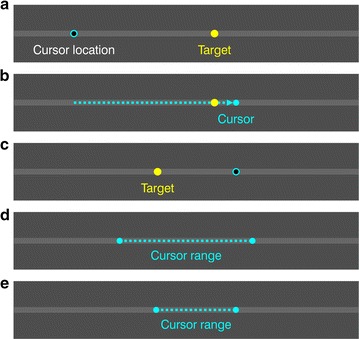
Visuomotor learning task. These five panels show a portion of the computer screen on which the target and cursor appear. **a** A target (*yellow dot*) appears at a random location on a fixed, *gray horizontal line* spanning the screen and centered on it vertically. The cursor is invisible and at a location (*cyan circle*) determined by the joystick position and the mapping between joystick and cursor. **b** Using the joystick, the subject moves (*dotted arrow*) the invisible cursor, trying to bring it to the target, and then shoots. Right after the shot, the cursor becomes visible (*cyan dot*) for 0.5 s, giving the subject feedback about their accuracy. **c** The cursor then vanishes again and the target jumps to a new, random location. The subject again tries to move cursor to target and shoot. Fifteen of these shots make up one test block. In each new block there is a new, random mapping, defined by a magnification factor *m* and a shift *s*, relating joystick position to cursor location. **d** For example, when *m* = 1 and *s* = 0 then a motion of the joystick across its entire range moves the cursor over the range shown here. **e** When *m* = 0.6 and *s* = 0.15 then the same set of joystick positions yields a different, smaller and shifted, set of cursor locations

Subjects used a joystick to steer a cursor to a target on a computer screen and then pressed a trigger to “shoot”. The target jumped randomly from place to place on a horizontal line (see Fig. 1 and “Methods” section). The cursor was invisible except for a period of 0.5 s after each trigger-press; i.e., subjects saw it only *after* they shot. Then the cursor disappeared and the target jumped to a new location, and this process repeated for a total of 15 targets, to make up one *block*. For each new block, the computer chose a new, random mapping relating joystick position to cursor position, so the subject had to relearn to make accurate shots. Each subject performed 30 of these 15-shot blocks: six warm-up blocks and then 24 test blocks which we used to analyze their learning.

**Fig. 2:**
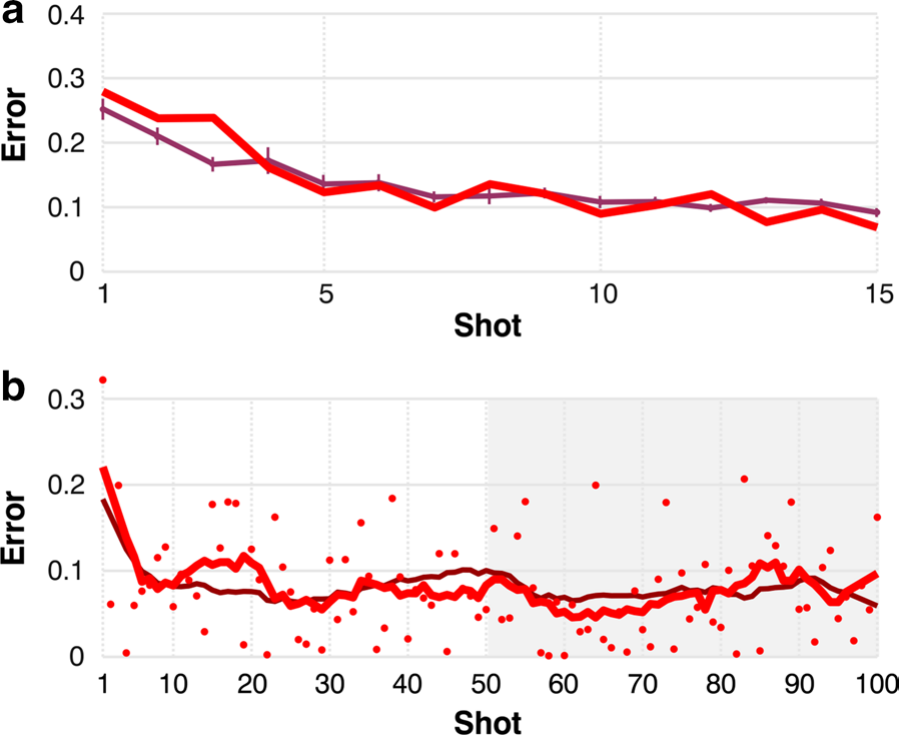
Human learning. **a** The *thick, bright red line* shows human subject 9’s unsigned error versus shot number, averaged over all 24 test blocks. The *thinner, dark red line* shows the shot-by-shot errors averaged across all 12 subjects; small vertical lines along this curve show standard errors of the mean, across subjects. **b** *Red dots* show the same subject’s errors on the 100-shot long block. The *thick, bright red line* is a running average of those errors, with a nine-shot sliding window. The *thinner, dark red line* is the mean running average across all subjects. We take each subject’s average error over shots 51–100 (*pale gray* interval) as a measure of their long-term accuracy

To quantify learning, we plotted unsigned shooting error (i.e., distance from cursor to target at the moment the trigger was pressed, averaged across all 24 test blocks) versus shot number. For example, the thick, bright red line in Fig. 2a shows the shot-by-shot errors for a typical subject (subject 9 of the 12). The thinner, dark red curve in the same panel is the average across all 12 subjects, and the thin vertical lines on this average curve show standard errors of the mean, across subjects.

All subjects showed the pattern in Fig. 2a, improving over the first several shots and then leveling off at a mean error well above zero. For our study it was immaterial whether learning was finished by the 15th shot, but we wanted to estimate our subjects’ long-term accuracy to help us program simulations. To that end, we had each subject do one *long block*, identical to the test blocks but with 100 rather than 15 shots. This long block always came right after the 14th test block. An example, for subject 9, is shown in Fig. 2b. Red dots show the shot-by-shot errors. The thick, bright red curve, a running average of those errors with a sliding window of nine shots, shows no discernible, consistent improvement after about 15 shots. The thinner, dark red curve, a running average of the aggregated data of all subjects, shows the same thing. We took each subject’s average error over shots 51–100 (the pale gray interval in Fig. 2b), as a measure of their *long*-*term accuracy*. Of course they might have improved further in the very long term, after thousands of trials, but this measure sufficed for our purposes, letting us program simulated learners that achieved humanlike accuracy on the time scale of our experiment, as described below.

### Simulated learners

We compared our humans to computer-simulated learners on the same task. These simulations received “visual” input (the target location) and responded with a “motor” output (a joystick position). Like the humans, the simulations tried to learn their joystick-cursor mappings, to improve their shooting. We call these simulations *doppelgängers* because they were duplicates of our individual humans; e.g., each doppelgänger had the same initial, pre-learning error rate as its human, and some of them were programmed with levels of response variability to make them match the long-term accuracy of their humans. Each human had three doppelgängers—a zeroth, a first, and a second-order learner—and each of these doppelgängers was optimized to learn its task as well as possible (given the response variability and initial performance it took from its human), so that if it lost to its human then the contest would be decisive. For instance, if people outperform the best zeroth-order learner then they cannot be relying on zeroth-order learning alone, but must be using first or second-order mechanisms.

**Fig. 3:**
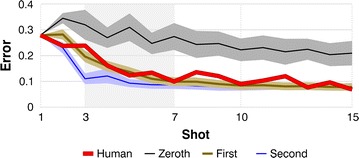
One human versus algorithms of all three orders of complexity. The *thick, bright red line* is the error curve for subject 9, reproduced from Fig. 2a. The *thin, black line* at the *top* of the graph is the mean curve for this subject’s zeroth-order doppelgänger, and the surrounding gray region shows its standard deviation. The *thin, blue line* at the *bottom* of the graph shows the same data for the subject’s second-order doppelgänger. The *brown line* that roughly coincides with the human curve represents the first-order doppelgänger. In most of our analyses, we compared the different learners’ mean errors on shots 3 through 7, the *pale gray* interval in the figure

First and second-order doppelgängers had Gaussian variability added to their motor responses so they matched the long-term accuracy of their humans (see “Methods” section). Without this added variability, these doppelgängers achieved near-perfect accuracy, very unlike human subjects, whereas with human-level variability, they showed much more human-looking performance, leveling off at nonzero error levels, as in Fig. 3. Zeroth-order doppelgängers had *no* variability added, because we wanted to show conclusively that humans outperform zeroth-order learners, even when the latter have the advantage of perfect precision.

Figure 3 shows subject 9′s error curve from Fig. 2a (the thick, bright red line), together with data from all three of this subject’s doppelgängers. All humans and doppelgängers did the same 24-block experiment, with the same 24 joystick-cursor mappings and the same sequences of 15 targets within each block. But the doppelgängers repeated the experiment one million times, their results varying because, for instance, the random perturbations used in the zeroth-order learning algorithm (see “Methods” section) varied from repetition to repetition. The thin, black line running near the top of Fig. 3 is the mean error curve for the zeroth-order doppelgänger, and the gray region around it marks plus and minus one standard deviation. The thin, blue line near the bottom of the figure represents the second-order doppelgänger in the same format. And the brown line in between, roughly coinciding with the human curve, represents the first-order doppelgänger.

### Humans versus zeroth-order learners

To compare humans and doppelgängers, we first examined each subject’s error curve (averaged across the 24 test blocks, as in Figs. 2a, 3) and recorded the mean height of this curve across the five shots 3 through 7—the pale gray interval in Fig. 3. This mean value we call the subject’s *early error*. We based our analysis on shots 3 through 7 because this is a period in which the three orders of learning algorithm are very distinct. At the start of each block all three have identical errors, and late in the block the slower algorithms are catching up to the faster ones, whereas over shots 3–7 the three orders are clearly separated.

**Fig. 4:**
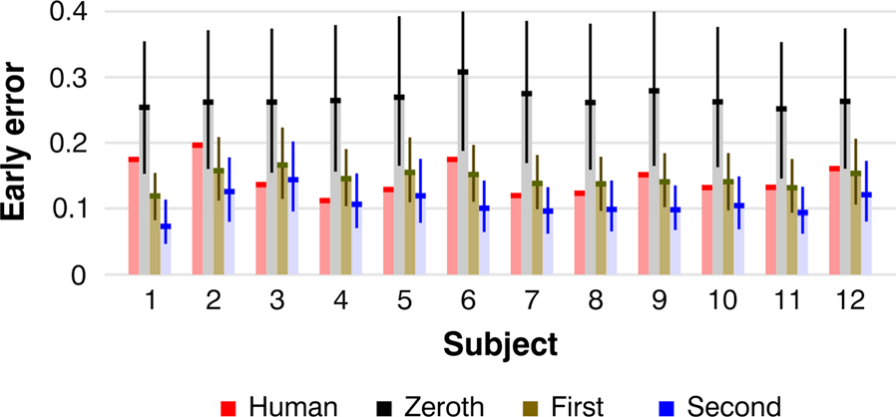
All humans versus algorithms of all orders. For each of the 12 subjects there is a cluster of four bars depicting, from *left* to *right*, results for the human (in *pink*), the zeroth-order doppelgänger (in *light gray*), the first-order (in *light brown*), and the second-order (in *light blue*). The *thick, red horizontal line* at the *top* of each *pink bar* show that human’s mean early error across the 24 test blocks. The *thick horizontal line* at the *top* of each doppelgänger bar shows its mean early error across one million repetitions of the whole 24-block experiment. The *thin vertical line* shows the range of results achieved by the doppelgänger over those million repetitions. The graph shows that humans outperformed zeroth-order doppelgängers, achieving smaller early errors; i.e., the leftmost, *pink bar* in each cluster is shorter than the adjacent, *light gray bar*. And humans learned worse than second-order doppelgängers, while contests with first-order doppelgängers yielded mixed results

Figure 4 compares all 12 human subjects’ early errors with those of their doppelgängers. Pink bars (leftmost bars in each cluster of four) show the human values. Pale gray bars (second from the left in each cluster of four) show the early errors on the same task for the zeroth-order doppelgängers. Again, each doppelgänger did the same experiment as the humans, but repeated it one million times. The thick black horizontal line at the top of each gray bar is the doppelgänger’s mean early error, averaged across the million repetitions. The thin vertical black line shows the range of early errors across those million tries. Comparing these ranges with the pink bars shows that for subjects 3 through 11, none of the million repetitions yielded an early error as small as the human’s, i.e., these nine humans significantly outperformed their zeroth-order doppelgängers, with *p* values <10^−6^. This brute-force method of computing *p* is more robust than t-tests or even most nonparametric tests, as it makes fewer assumptions about data distributions. The remaining subjects (1, 2, and 12) were occasionally surpassed by their zeroth-order doppelgängers, but only 208 times out of one million tries for subject 1, 4558 out of a million for subject 2, and once for subject 12, i.e., their *p* values were 2.08 × 10^−4^, 4.558 × 10^−3^, and 10^−6^, respectively. These results show that all 12 humans significantly outperformed their zeroth-order doppelgängers.

As one would expect, given these individual statistics, humans also outperformed zeroth-order doppelgängers when tested as a group. That is, we repeated the experiment one million times on all the zeroth-order doppelgängers and for each repetition we computed the mean early error averaged across those 12 doppelgängers, and compared that value to the humans’ mean early error, averaged across the 12 subjects. The doppelgängers achieved a lower value than the humans’ in none of those million tries; i.e., the *p* value was less than 10^−6^. We conclude that the humans’ learning did not rely solely on zeroth-order mechanisms.

### Humans versus first and second-order learners

When we compared humans to first-order doppelgängers (Fig. 4, light brown bars, the third bar in each cluster of four), the results were mixed and not statistically significant. Six of the 12 humans outperformed their doppelgängers, three of them with *p* values less than 0.01. The other six lost to their doppelgängers, and again three of them had *p* < 0.01. As a group, humans did not differ significantly from these doppelgängers (*p* = 0.421). In short, we found no significant difference between humans and first-order doppelgängers. In particular, we found no evidence for the one result that would have had clear implications for the order-of-complexity question: our humans did not defeat their optimized first-order doppelgängers, and therefore we cannot say whether human learning incorporates second-order mechanisms.

Compared to second-order algorithms (light blue bars, rightmost in each cluster of four), humans learned significantly worse. Eleven of the 12 subjects lost to their second-order doppelgängers, and no subject significantly outperformed the doppelgänger. Specifically, subjects 1, 2, and 6–12 lost to their second-order doppelgängers, with *p* values ranging from 0.0008 to less than 10^−6^. Subjects 4 and 5 also performed worse than the mean of their second-order doppelgängers, though not significantly, with *p* values of 0.24 and 0.15. Subject 3 learned better than the mean of his doppelgänger, but not significantly, with *p* = 0.26. As a group, humans learned more slowly than second-order doppelgängers, with a *p* of less than 10^−6^.

### Other tests

When we analyzed male and female subjects separately, they showed the same pattern. They both defeated their zeroth-order doppelgängers, in both cases with *p* values less than 10^−6^; and they both lost to the second-orders, both again with *p* values less than 10^−6^.

We found no evidence of meta-learning during our experiment. Comparing early errors in the first six versus the final six test blocks, we found no difference, with *p* = 0.569 by the signed rank test. There was improvement between the six *warmup* blocks and the final six test blocks, with *p* = 0.0004 by the signed rank test, but the warmup blocks were not included in the analysis. In any case, our experiment was designed so that meta-learning, even if it had occurred, would not have affected our conclusions, because the doppelgängers already had all the advantages that humans might gain through meta-learning (see “Discussion” section).

We tested the robustness of our main results by looking at two possible sources of variation. First, our analyses in Figs. 3 and 4 were based on early error, over shots 3 through 7. We chose that interval for a good reason, as described above, but was that choice critical? Specifically, do our findings change when we redo the analyses separately for each shot after the first, checking, for instance, whether humans achieve significantly lower errors than zeroth-order doppelgängers on shot 2, on shot 3, and so on? Second, in Figs. 3 and 4 we added variability to our first and second-order doppelgängers so they matched the long-term accuracy of their humans, but our estimates of human long-term accuracy may not have been exact. How do our findings change when we alter doppelgänger variability?

In these tests of robustness, the zeroth-order results were perfectly consistent. Zeroth-order doppelgängers lost significantly to humans (with *p* values less than 10^−4^) in all 14 tests, at every shot from 2 through 15. Second-order results were also consistent. Second-order doppelgängers defeated their humans at every shot from 2 through 15, and did so significantly, with *p* < 0.01, at shots 2 through 11. At shot 12, *p* was 0.044, and at shot 15 it was 0.156, rising, as one would expect, because the difference between second-order and slower algorithms shrinks as the slower ones catch up, later in the block. And second-order doppelgängers still defeated the humans over shots 3 through 7 even when each doppelgänger’s variability was increased by 35%. (At shot 11 and beyond, these highly variable doppelgängers *lost* resoundingly to the humans, with *p*’s of 10^−3^ or less, as one would expect, because their added variability made their long-term accuracy markedly worse than the humans’). In short, our zeroth and second-order results were robust.

Comparisons of humans and first-order doppelgängers yielded mixed and inconclusive results in Fig. 4, and they did so again in our tests of robustness. For example, first-order doppelgängers lost to their humans at shots 2, 3, and 5, but defeated them at all other shots. And they were more sensitive to variability than were the second-order learners; e.g., when we raised their variabilities by 20%, they defeated their humans on only 2 shots out of 14. In brief, these comparisons were again inconclusive, and in particular they yielded no evidence that humans outlearned optimized, first-order doppelgängers.

## Discussion

The terms *zeroth*, *first*, and *second*-*order* come from optimization theory, of which the theory of learning algorithms is a branch. Second-order algorithms are ones that compute the second derivative of the error (or *loss* or *risk*, whatever we call the quantity to be minimized) with respect to the learner’s adjustable parameters. First-order methods compute the first but not the second derivative. And zeroth-order methods compute neither.

A simple way to distinguish zeroth-order mechanisms from the other classes is that zeroth-order methods work with an unsigned, scalar error signal, rather than a signed error, e.g., distance from cursor to target, rather than signed distance. Signed errors of course carry more information than unsigned errors, but exploiting that information requires more complex processing. Therefore, zeroth-order learning is simpler than the other kinds, and easier to implement in neural networks. It requires only a single scalar feedback signal, which can be “broadcast” to all the neurons in a network [9], whereas in first or second-order algorithms, feedback is vectorial and is usually distributed in more complicated ways [16–18]. Many of these distribution schemes have been deemed biologically implausible [17–23], though others *are* feasible [11, 13, 24, 25].

Zeroth-order methods are attractive also because they require fewer assumptions. That is, they need less prior information about the structure of the neural network, its neurons, and the environment.

And finally, zeroth-order methods are interesting for *motor* learning, specifically, because they provide an easy solution to the *distal teacher* problem, which is central to motor control [13, 14, 26]. The issue here is that, in most artificial neural networks, there is a simple, known relation between the network’s output ***y*** and the error signal ***e*** that guides its learning, e.g., we might have ***e*** = ***y*** − y*, where y* is desired output. But in motor learning, the output of the network is motoneuron firing, ***u***, while error signals are sensory; e.g., you see or feel that your hand came down to the left of its target. The relation between ***u*** and ***e*** depends on many intervening factors, such as sensor properties and the mechanics of joints and muscles. To learn in this setting, derivative-based mechanisms (i.e., first or second-order algorithms) must know the derivative of ***e*** with respect to ***u***, and getting that knowledge calls for additional mechanisms or assumptions [13, 14, 26, 27]. But zeroth-order algorithms do not need to know ∂***e***/∂***u***. They simply try out random parameter changes, monitor the results, and accept those changes that improve performance. In other words, zeroth-order mechanisms need no additional circuitry to operate in a motor-control setting.

But our results show that humans outperform optimized zeroth-order doppelgängers, and therefore human visuomotor learning cannot rely on zeroth-order mechanisms alone. Of course zeroth-order processes may still be operating in the brain, but they cannot be the *sole* mechanism of learning. First or second-order mechanisms must be contributing as well.

Compared to second-order algorithms, we found that humans learned worse. This finding shows that humans do not use perfectly optimized second-order methods, but leaves open the possibilities that they used suboptimal or approximate second-order mechanisms, or a mix of second and lower-order elements.

Comparisons of humans and first-order doppelgängers yielded results that were mixed and inconclusive. In particular, if our humans had outperformed their optimized, first-order doppelgängers, then we could have concluded that human learning incorporates second-order mechanisms; but given our results, that remains an open question.

Overall, then, our results show that visuomotor learning uses *at least* first-order mechanisms. That finding is consistent with numerous papers in the literature which posit first-order (i.e., gradient-based) learning mechanisms in the brain, e.g., [11–15].

We found no evidence of meta-learning during our experiment. And in any case, meta-learning, even if it had occurred, would not have affected our analysis or conclusions, as it would not have given the humans any advantage over their doppelgängers. Meta-learning would mean that subjects improved their joystick precision, their hyperparameters such as the learning rate parameter η, or their hypothesis spaces (i.e., they got accustomed to the ranges of the joystick-cursor relations used in the experiment). But the zeroth-order doppelgängers were already optimized in all these respects: they had perfect precision, optimized hyperparameters, and hypothesis spaces (see “Methods” section) ideally suited to the task. First and second-order doppelgängers did not have perfect precision, but their precision was matched to the human data, and so any improvement in the humans would have been passed on to those doppelgängers.

Our key finding is that human learning is not purely zeroth-order. We ruled out pure zeroth-order learning in just one, extremely simple visuomotor task, but that is enough to show that at least some human learning incorporates first or second-order mechanisms. And purely zeroth-order methods are even less likely in complex, nonlinear, high-dimensional tasks than in simple ones, because they get slower and slower, relative to first and second-order mechanisms, as the number of adjustable parameters grows [1, 24].

## Conclusion

Human visuomotor learning does not rely on zeroth-order mechanisms alone, but uses at least first-order mechanisms and maybe second-order as well.

## Methods

### Human subjects

Subjects were 12 healthy volunteers, six male and six female, aged 20–29.

### Joystick

Subjects shot at targets using a joystick—a Thrustmaster T.16000M (Guillemot Corporation, France), for which the range of hand motion was 13.6 cm both horizontally and vertically, though in our experiments only horizontal motion affected the motion of the cursor on the computer screen. Rightward motion of the joystick moved the cursor to the right on the screen, and leftward motion moved it left.

### Target and cursor

The target was a yellow disk of diameter about 3 mm, or 0.3° of visual angle, on a computer screen 55 cm from the subject’s face. The subject tried to steer a cursor to the target and then press a trigger to “shoot”. The cursor was invisible except that after each shot it appeared for 0.5 s, in the form of a cyan disk about 0.25° across. Then the cursor disappeared again and the target jumped to a new, random location (chosen from a distribution described below). The invisible cursor did not jump anywhere—it was always in whatever location was determined by the joystick. The cursor was programmed to ignore vertical motion of the joystick, so that it stayed on a horizontal line centered on the computer screen, and the target also appeared always on that same line. If the subject did not shoot within 1.5 s of the target appearing then the target blinked, as a reminder to keep up a quick pace; mean response time varied from subject to subject, ranging from 0.74 to 1.28 s. All software for the experiments, data recording and analysis was written in Matlab by the authors.

### Blocks

Each subject performed 30 blocks of 15 shots each: six warmup blocks followed by 24 test blocks which we used in our analysis. Between the 14th and 15th test blocks, each subject performed one long block of 100 shots. Subjects were not told that any of the blocks were warmups; they simply did twenty 15-shot blocks, one 100-shot block, and another ten 15-shot blocks.

### Joystick-cursor mapping

For each new block, the program chose a new, random mapping relating joystick to cursor. Specifically, joystick position determined cursor position (not velocity) by a *magnification* factor *m* chosen from the range 4/7 to 4, and a *shift s* from the range −1 to 1 (the reasons for these ranges, and the procedure for randomly selecting *m*’s and *s*’s, are described below, under Policy). Therefore cursor location *c* was related to joystick position *j* by the equation *c* = *mj* + *s*. Here *s* = 0 meant that the cursor was at the center of the screen when the joystick was at the center of its range, and *m* = 1 meant that a maximal, 13.6-cm motion of the joystick, from its leftmost to its rightmost extreme, moved the cursor 10.6 cm on the computer screen; i.e., from −1 to 1 in the screen coordinates defined below.

### Doppelgängers

We compared our humans to simulated learners programmed in Matlab. We call these simulations *doppelgängers* to emphasize that they were duplicates of our individual humans, in three respects. First, each doppelgänger underwent the same *testing* as its human: the same sequence of 24 joystick-cursor mappings, and the same sequences of 15 targets in each block. Second, each doppelgänger went into each block with an initial hypothesis about the joystick-cursor mapping that matched the *initial errors* of its human. From each human’s 24 first-shot errors, we computed an initial weight and bias, *w*_0_ and *b*_0_, such that doppelgängers that began each block with *w* = *w*_0_ and *b* = *b*_0_ showed the same mean, unsigned error on shot 1 as that human; e.g., in Fig. 3, subject 9’s doppelgängers all show the same initial error as their human. Finally, some doppelgängers (the first and second-order ones) were programmed with a response variability so they matched the long-term accuracy of their humans (see “Response variability” section, below).

### Optimization

In these ways, each doppelgänger was matched to its human. But in all other ways it was *optimized* to learn its task as well as possible. Doppelgängers were optimized in three ways. First, of the many algorithms in each class (zeroth, first, and second-order), they used the fastest one, based on our own tests and the literature: for zeroth-order, they used node perturbation [3]; for first-order, the LMS algorithm [4, 5, 28]; and for second-order, RLS [6, 28, 29]. Second, each doppelgänger used the hyperparameters (see below) that yielded the smallest mean end-of-block error on its task. So each doppelgänger was optimally suited to this task in every detail—the ranges of mappings and targets, the number of shots per block, and its own response variability. It is very unlikely that humans’ learning networks are so perfectly suited to this specific task. Third, each doppelgänger was equipped with an optimal *hypothesis space*. Here the idea is that no learner can learn every possible pattern. There is always some limited repertoire, or hypothesis space [30]. If you have a larger repertoire then you can learn more patterns, but you learn more slowly because you have to search through a larger set of possibilities [30]. For the best results on any one task, you want the smallest hypothesis space that nonetheless contains all the patterns you will have to learn. Accordingly, our doppelgängers had hypothesis spaces that allowed only magnify-and-shift mappings, with a magnification factor *m* in the range 4/7–4 and a shift *s* in the range −1 to 1—much narrower than any human’s repertoire, and coinciding perfectly with the shifts and magnifications used in the task. These design choices weighted the human-doppelgänger contests in favor of the doppelgängers.

### Policy

Owing to their optimized hypothesis spaces, all doppelgängers of all orders knew that the joystick-to-cursor mapping had the form *c* = *mj* + *s*. Therefore, they knew that the optimal policy (i.e., the rule for choosing a joystick position *j* to make cursor location *c* match target location *x*) was *j* = (*x* − *s*)/*m*. But of course the specific *m* and *s* used in each block were random and unknown to the doppelgänger, as they had been to its human counterpart. Therefore, doppelgängers of all orders used policies of the form *j* = *wx* + *b*, where *w* and *b* were an adjustable *weight* and *bias* which the doppelgänger adjusted by learning, trying to drive *w* toward 1/*m* and *b* toward −*s*/*m*. In other words, for each block there was an optimal weight w* = 1/*m*, and an optimal bias b* = −*s*/*m*, and the learning algorithms tried to drive *w* to w* and *b* to b*. For each block, the computer running the experiment chose w* randomly from a uniform distribution over the range 0.25–1.75, and b* from a uniform distribution over −0.25 to 0.25, and from those values computed the *m* and *s* factors for the block (and as a result, *m* and s varied over the ranges 4/7–4 and −1 to 1).

All our doppelgängers had policies of this form, with only two adjustable parameters, *w* and *b*. Each doppelgänger can therefore be represented as a very simple “network”, of just a single neuron, receiving a single input (the target location *x*) and applying a weight and bias to compute a motor command of the form *j* = *wx* + *b*. (The complete equations for these networks’ learning rules are laid out in the three sections, *Zeroth*, *First*, and *Second*-*order simulations*, below.) Human brains, in contrast, are huge, deep networks. Does this fact give the humans an advantage; i.e., could a zeroth-order learner match or defeat a human after all, if it computed *j* using a large, multilayer network?

It could not. Larger networks do not learn our mapping task any faster or more accurately than small ones. Large networks, with many adjustable parameters, are inherently *slower* learners than small networks, because they have to search through a higher-dimensional space of possible parameter settings [1, 5, 30]. The fastest learning is achieved by using the simplest network that is capable of representing the patterns to be learned. That is why our doppelgängers have exactly two adjustable parameters, *w* and *b*, rather than millions, and are capable of representing *only* magnify-and-shift mappings. The complex networks in human brains actually put us at a slight disadvantage in this task. Our complexity is of course hugely advantageous for *other* tasks, and it makes us versatile—able to learn a wide array of motor skills. But if the only things we ever had to learn were one-dimensional magnify-and-shift mappings, then a single neuron with two adjustable parameters would be best. So it is very unlikely that any more-complex zeroth-order learners could outperform our simple, optimized ones on this task. We have confirmed that fact with simulations of more-complex doppelgängers (not shown), though these simulations also confirm that large networks, appropriately structured, are only *slightly* slower than simple ones on our task (e.g., early error is about 1.5% higher in zeroth-order learners with 100 or 1000 neurons instead of one).

It is also true that the brain has mechanisms—such as pruning, axon growth, attention, and habituation—which let it simplify and reshape its large networks, or perhaps focus their processing on smaller subnetworks, and so mitigate the problem that large nets learn more slowly than small ones. These mechanisms are important in real brains and absent from our doppelgängers, but again they do not affect our conclusions, i.e., they do not put the doppelgängers at a disadvantage relative to the humans. The reason, as in our earlier discussion of meta-learning, is that the advantages provided by these mechanisms are built into the doppelgängers from the beginning. That is, pruning, attention, and other processes can simplify neural networks, but our doppelgängers are provided from the start with the simplest possible network architecture compatible with their learning task.

### Screen coordinates

We defined the leftmost joystick position as *j* = −1 and the rightmost as *j* = 1. Given that cursor location *c* = *mj* + *s* = (*j* − b*)/w*, and that the minimum values of w* and b* were 0.25 and −0.25, it follows that the maximum possible *c* in the experiment was (*j*_max_ − b*_min_)/w*_min_ = (1 − (−0.25))/0.25 = 5, and the minimal *c* was −5. Therefore we adopted a coordinate system for the 53-cm wide computer screen in which the leftmost edge was −5 and the rightmost was 5.

### Target distribution

We ensured that all targets appeared within a central region of the computer screen, small enough that every target in every block was reachable by the cursor, regardless of that block’s magnification and shift. Further, we used the same target range in all blocks, regardless of the current *m* and *s*, so that the target locations offered the subject no clues about w* and b*. Given that *c* = (*j* − b*)/w*, and the maximum possible values of *j*, w*, and b* in the experiment were 1, 1.75, and 0.25, it follows that the rightmost target location *x* that is reachable by the cursor when both w* and b* are maximal is *x* = (*j*_max_ − b*_max_)/w*_max_ = (1 − 0.25)/1.75 = 0.4286. Similarly, the leftmost *x* that is always reachable, regardless of w* and b*, is −0.4286. Therefore all targets for all blocks were chosen randomly from a uniform distribution over the range −0.4286 to 0. 4286 in screen coordinates. This target range spanned 4.56 cm at the center of the screen, or about 4.75° of visual angle. So it was small, but clearly visible, and none of our subjects reported any difficulty with it; and once again, if any subjects *had* experienced difficulty with the target range or any other aspect of the task, it would not have weakened our conclusions, because if anything it would have impaired human performance, not given the humans an advantage over their doppelgängers.

### Zeroth-order simulations

Zeroth-order doppelgängers used a node perturbation learning algorithm [3]. The learner produced, for each shot, a motor response *j* = *wx* + *b* + *g*, where *j* was joystick position, *x* was the target location, and *g* was a random, Gaussian perturbation with a mean of 0 and standard deviation σ_*g*_. The signed error for that shot was *e* = *c* − *x* = *mj* + *s* − *x*, and the squared error or *loss* was *L* = *e* ^2^. The doppelgänger updated its *w* and *b* after each shot by the learning rules *w* ← *w* − η(*L* − *L*_*prev*_)*gx* and *b* ← *b* − η(*L* − *L*_*prev*_)*g*, where η was the learning rate constant, and *L*_*prev*_ was the loss on the previous shot. Then, also after every shot, the doppelgänger readjusted its *w* and *b*, if necessary, to keep them in the correct ranges, 0.25–1.75 and −0.25 to 0.25, respectively. In other words, the learner used its prior knowledge of the ranges of w* and b* to improve its estimates.

### Hyperparameters

Before testing the doppelgänger against its human, we used the Nelder–Mead algorithm to find its optimal hyperparameters σ_*g*_ and η; i.e., the values that yielded the smallest mean error on shot 15, at the end of the block, given all the details of the visuomotor task: the ranges of mappings and targets, the number of shots per block, and the start-of-block accuracy of that doppelgänger’s human. These optimal σ_*g*_’s and η’s were used in the simulations in Figs. 3 and 4. We also tested zeroth-order doppelgängers with *three* optimized hyperparameters: σ_*g*_ and two separate learning rate constants, η_w_ and η_*b*_, and learning rules *w* ← *w* − η_*w*_(*L* − *L*_*prev*_)*gx* and *b* ← *b* − η_*b*_(*L* − *L*_*prev*_)*g*, but the results (not shown) were virtually identical, with the doppelgängers again losing to their humans with *p* < 10^−6^.

### Response variability

Human joystick responses were highly variable, as is evident in Fig. 2b, and this variability limits the accuracy that human subjects can attain in our visuomotor task. Doppelgängers, in contrast, can be programmed to have perfect precision, but doing so gives them an unphysiological advantage over the humans, letting first and second-order doppelgängers quickly achieve mean errors very near zero. We therefore added human-level variability to those doppelgängers, as described below.

### First-order simulations

Each first-order doppelgänger had a Gaussian random variable *r* added to its motor outputs; i.e., *j* = *wx* + *b* + *r*, where *r* had a standard deviation σ_*r*_, set so that the doppelgänger’s median unsigned error over the final 50 shots of a 100-shot long block matched that of its human under the same conditions—the same 100-shot block with the same w* and b* and targets. The mean σ_*r*_, averaged across subjects, was 0.0705; the range, 0.0481–0.0942. (As we have seen, zeroth-order doppelgängers also had a random variable, *g*, that affected *j*, but those *g*’s were perturbations deliberately applied to *j* and used in the zeroth-order learning algorithm, and their standard deviation σ_*g*_ was chosen to optimize learning, whereas each first-order doppelgänger’s random variable, *r*, represented response variability, and its σ_*r*_ was chosen to match the behavior of its human—it was not set to the value σ_*r*_ = 0, which would have optimized learning.)

First-order doppelgängers learned using the least mean squares, or LMS, algorithm [5]. It is not possible to use LMS directly to adjust the weight and bias *w* and *b*, because you cannot compute the relevant derivatives unless you already know w* and b*. (And of course our human subjects and doppelgängers did not know these things initially—the whole point of their learning was to find w* and b*. This difficulty with using LMS directly on the policy parameters *w* and *b* is a consequence of the distal teacher problem, described briefly in our Discussion and in depth elsewhere [13, 26]). The solution was to have the doppelgängers run LMS on the magnification and shift variables *m* and *s*. Each doppelgänger began each block with an initial estimate of the magnification for that block, *m*_est_ = 1/*w*_0_, and an initial estimate of the shift, *s*_est_ = −*b*_0_/*w*_0_, where *w*_0_ and *b*_0_ were the individualized initial weights and biases described earlier. Then, after each shot, it adjusted those estimates by the LMS learning rule, *m*_est_ ← *m*_est_ − η_*m*_ *ej* and *s*_est_ ← *s*_est_ − η_*s*_ *e*, where η_*m*_ and η_*s*_ are learning rate constants and *e* is the signed error, *e* = *c* − *x*. Based on these estimates, it then updated its weight and bias, *w* = 1/*m*_est_ and *b* = −*s*_est_/*m*_est_. And like the zeroth-order learners, these first-order ones then adjusted *w* and *b*, if necessary, to keep them in the correct ranges, and made corresponding corrections to *m*_est_ and *s*_est_. For first-order as for zeroth-order doppelgängers, we found the optimal η’s using the Nelder–Mead algorithm.

### Second-order simulations

Second-order doppelgängers, like first-orders, had Gaussian variability added to their motor commands to give them the same long-term accuracies as their humans. They learned using the recursive least squares, or RLS, learning algorithm [6], which involves a hyperparameter μ in the range 0–1, and several new variables: vectors ***y***, ***v***, and ***k***, and a matrix ***P*** initialized to the identity, ***P*** = ***I***. After each shot, these doppelgängers computed ***y*** = (*j*, 1)^T^, ***v*** = ***Py***, ***k*** = ***v*** ^T^/(μ + ***y*** ^T^ ***v***), ***P*** ← ***P*** − ***vk***, *m*_est_ ← *m*_est_ + *ek*_1_, *s*_est_ ← *s*_est_ + *ek*_2_, *w* = 1/*m*_est_, *b* = −*s*_est_/*m*_est_. For each doppelgänger, we used the Nelder–Mead algorithm to find the hyperparameter μ that was optimal for its task.

## Additional file

**Additional file 1.** An .xls file containing all the raw data from the study. The file has 12 pages—one for each of the 12 subjects. Each page shows all of its subject’s data: the 30 blocks of 15 targets each, and the long block of 100 targets. For each block, the page shows the w* and b* values for that block, the time of each shot (since the start of the block), the target location for that shot, and the cursor location when the shot was taken.
